# Anti-Inflammatory Effects of GLP-1 Receptor Activation in the Brain in Neurodegenerative Diseases

**DOI:** 10.3390/ijms23179583

**Published:** 2022-08-24

**Authors:** Yolanda Diz-Chaves, Zainab Mastoor, Carlos Spuch, Lucas C. González-Matías, Federico Mallo

**Affiliations:** 1 Biomedical Research Centre (CINBIO), University of Vigo, Galicia Sur Health Research Institute, 36310 Vigo, Spain; 2 Translational Neuroscience Research Group, Galicia Sur Health Research Institute, CIBERSAM, Hospital Álvaro Cunqueiro, Bloque Técnico, Planta 2, Sala de Investigación, Estrada Clara Campoamor, 341, 36212 Vigo, Spain

**Keywords:** GLP-1, GLP-1 RAS, GLP-1R, brain inflammation, multiple sclerosis, ALS, Alzheimer’s disease, Parkinson’s disease, microglia, astrocytes

## Abstract

The glucagon-like peptide-1 (GLP-1) is a pleiotropic hormone well known for its incretin effect in the glucose-dependent stimulation of insulin secretion. However, GLP-1 is also produced in the brain and displays a critical role in neuroprotection and inflammation by activating the GLP-1 receptor signaling pathways. Several studies in vivo and in vitro using preclinical models of neurodegenerative diseases show that GLP-1R activation has anti-inflammatory properties. This review explores the molecular mechanistic action of GLP-1 RAS in relation to inflammation in the brain. These findings update our knowledge of the potential benefits of GLP-1RAS actions in reducing the inflammatory response. These molecules emerge as a potential therapeutic tool in treating neurodegenerative diseases and neuroinflammatory pathologies.

## 1. Introduction

The idea of considering the central nervous system (CNS) as an immune-privileged site is outdated now [1]. Immune cells are present in the meninges and provide immune surveillance of the CNS [1]. Although there are no antigen-presenting cells within the CNS parenchyma, this role can be performed by dendritic cells at the interface of the CNS and the immune system in association with blood vessels [2]. Moreover, the blood-brain barrier is part of the innate immune defense against pathogens entering the CNS [3,4]. Two cell types: microglia and astrocytes are involved in the immune defense in the parenchyma [4].

Microglia are the principal resident immune cells of the brain and are involved in homeostasis and host defense against pathogens and CNS disorders [5]. In response to injury or disease, microglia undergo a process of cellular activation. This is characterized by phenotypical morphological changes, an increase in cell proliferation, and modifications in their function [6], leading to proinflammatory production mediators [7,8,9,10]. Microglia are the first cells to respond to CNS insults, followed by reactive astrocytosis [11]. Microglia cells also have a prominent role in the etiology and progression of different neurodegenerative diseases [8].

Astrocytes are involved in nearly every aspect of the CNS function. This includes synapse formation and maturation, ion and neurotransmitter homeostasis, blood-brain barrier maintenance, and neuronal metabolic support [12,13]. Astrocytes’ activation may be beneficial, promoting tissue repair and homeostasis, or detrimental, exacerbating inflammatory reactions and tissue damage, depending on the stimuli’ nature [14,15].

Inflammatory processes influence most neurological disorders, even those whose primary etiology is not inflammatory [16]. They are related to several causes: protein aggregates, accumulation of other abnormally modified cellular constituents, molecules released from injured neurons or synapses, and dysregulation of inflammatory control mechanisms [14]. In this regard, chronic brain inflammation is involved in the etiopathology of many neurodegenerative diseases such as Alzheimer’s disease (AD), Parkinson’s disease (PD), multiple sclerosis (MS), and amyotrophic lateral sclerosis (ALS); but also, in neuropsychiatric disorders (schizophrenia, autism spectrum disorders, depression, bipolar disorder, among others), epilepsy, degenerative diseases of the retina and optic nerve, stroke or traumatic brain injury (TBI) [17,18,19].

However, the immune system can be modified in several ways. It is a significant target for developing treatment strategies, opening a therapeutical window of opportunity to change many of these disorders with similar approaches [20]. The glucagon-like peptide (GLP-1) receptor agonists emerge as potent compounds, with immunomodulatory and anti-inflammatory properties that have been used in several diseases associated with chronic inflammation [21,22,23].

In this review, we have summarized the recent advances known about the anti-inflammatory effects of GLP-1 receptor activation in the brain in neurodegenerative diseases, especially in regards to microglia and astrocytes.

## 2. Glucagon-like Peptide 1 and the GLP-1 Receptor Agonist

The GLP-1 is a versatile hormone with broad pharmacological potential [24,25]. It is best known for its incretin effect in inducing insulin secretion in hyperglycemic conditions, but it also displays several metabolic effects [25]. GLP-1 decreases gastric emptying, inhibits food intake, increases natriuresis and diuresis, modulates rodent β-cell proliferation, regulates the hypothalamic-pituitary-adrenal (HPA) axis in rats, and has cardio- lung- and neuroprotective effects [25,26,27,28,29,30,31]. Moreover, GLP-1 modulates reward behavior and palatability, and regulates learning and memory [32,33,34].

**Figure 1:**
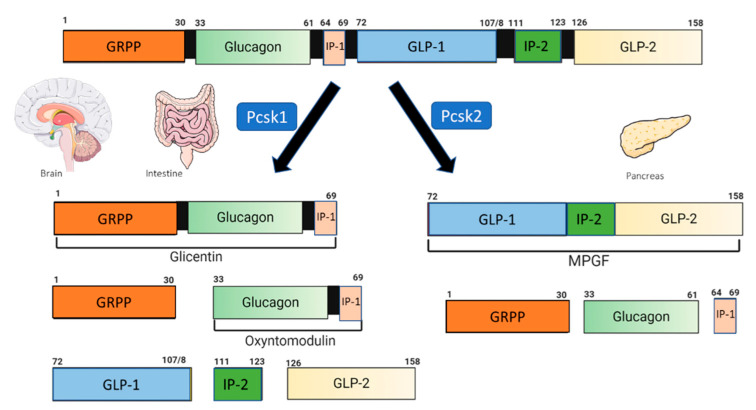
Schematic processing of proglucagon. NTS: nucleus of the solitary tract; GRPP: glicentin-related polypeptide; IP-1: intervening peptide-1; IP-2: intervening peptide-2; GLP-1: glucagon-like peptide-1; GLP-2: glucagon-like peptide-2; MPGF: major proglucagon fragment; PCSK1: prohormone convertase 1/3; PCSK2: prohormone convertase 2. Modified from [36]. Created with Biorender.com.

GLP-1 is synthesized from the proglucagon gene after cell-specific post-translational processing by two members of the convertase subtilisin/kexin (PCSK) family (Figure 1) [35,36]. The L-cells secrete GLP-1 at low basal levels in the fasting or interprandial state [37], and predominantly express the prohormone convertase 1 (PC1/3; Pcsk1). In addition to GLP-1, proglucagon cleavage results in the liberation of glucagon-like peptide two (GLP-2), oxyntomodulin, glicentin and intervening peptide-2 (IP2) [36,38]. Pancreatic α-cells primarily express PC2 (Pcsk2), with low expression of PC1/3. They produce glucagon, glicentin-related pancreatic polypeptide (GRPP), the significant proglucagon fragment (MPGF), and intervening peptide-1 (IP-1) [36,39]. In the intestine and to a lesser extent in the pancreatic α-cells, the processing of the proglucagon results in the formation of at least three GLP-1 peptides of 37, 31, and 30 residues [36]. The two N-terminally truncated products, a glycine extended peptide GLP-1(7–37) and GLP-1(7–36) amide, are recognized by the pancreatic receptor, and are the active species in vivo [40]. However, in vitro studies have demonstrated that its metabolite, GLP-1(9–36), has protective effects in neuronal and microglial cell lines [41].

The primary source of endogenous GLP-1 within the brain is a population of preproglucagon (PPG) neurons in the caudal portion of the nucleus of the solitary tract (NTS). It is synthesized after cell-specific post-translational processing by the prohormone convertases (Pcsk1) (Figure 1) [42,43]. The PPG cell bodies were also described in the adjacent medullary reticular formation in a population of glutamatergic olfactory bulb interneurons, the piriform cortex, and the lumbar-sacral spinal cord [44,45,46,47,48,49]. The NTS PPG neurons project to GLP-1 receptor (GLP-1R) expressing regions, with the hypothalamus being the primary target [36]. Other areas of the brainstem that control the autonomic function including the limbic forebrain regions [36,48,50], areas of the mesolimbic reward system related to control of feeding and motivation to feed [36,51,52], or structures implicated in the regulation of the stress response and the HPA axis, learning and memory [36,53], all receive neuronal projections from neurons of the NTS in rodents.

The short half-life of GLP-1 is limited in its therapeutic use, since it is rapidly metabolized and inactivated by the dipeptidyl peptidase IV after release to the extracellular space [25,54]. Different pharmacological agents, GLP-1 derivatives, with extended half-life and improved bioavailability, have been developed in the last few years for treating type 2 diabetes and obesity [25,55,56]. These molecules bind to GLP-1R and activate downstream signaling effects on cell growth and repair equivalent to GLP-1 [57].

There is currently 7 GLP-1 R agonist (GL1-RAs) approved for the treatment of type 2 diabetes [58]. GLP-1 analogs can be classified as: (i) Protection from DPP-IV cleavage based on the exendin-4 peptide (a naturally occurring peptide obtained from the venom of the “Heloderma lizard”), that bears a 53% homology to human GLP-1, and is resistant to DPP-4 inactivation [36]. Exenatide (Byetta^®^ and Bydureon^®^, AstraZeneca, Cambridge, UK), a synthetic 39-amino acid peptide that was the first GLP-1 RA approved for clinical use [25,59,60] or Lixisenatide (Sanofi, Paris, France), is an analog of exenatide, with comparable pharmacokinetic properties [25,61]. This group also includes semaglutide (Novo Nordisk, Copenhagen, Denmark), dulaglutide (Trulicity; Eli Lilly & Co., Indianapolis, IN, USA), and albiglutide (GlaxoSmithKline, London, UK) [62]. Albiglutide is covalently fused to human albumin, enhancing proteolytic stability and simultaneously delaying renal elimination [62]. (ii) Analogs of human GLP-1 in which the renal excretion slows down by conjugating the peptide to substances such as fatty acids (among others). Like Liraglutide (Novo Nordisk, Copenhagen, Denmark), it is based upon the native GLP-1 sequence. This GLP-1 RA is palmitoylated (C16:0) at the side chain of lysine 20 via a γ-glutamic acid spacer, prolonging the half-life for 13 h [25,36,59]. Semaglutide (Novo Nordisk) is a chemically-optimized analog of Liraglutide with enhanced pharmacological properties [25,36,62]. (iii) Linkage of two GLP-1 molecules via the Fc fragment of a monoclonal antibody (IgG4), as was applied for dulaglutide [62,63]. 

In addition, several structurally related hormones can be integrated into a single entity of enhanced potency by combining some sequences, enhancing potency, and sustained action [25]. The multi-agonists are characterized by a single molecule with complementary signaling through more than one receptor, each of which offers beneficial effects on systems [25]. This is the case of a dual or triple agonist of the GLP-1 receptor that includes glucagon or GIP (glucose-dependent insulinotropic polypeptide) [25], like MAR709 or Tirzepatide (a GLP-1R/GIPR dual-agonist peptide) [58].

Recently, small molecules’ positive allosteric modulators (PAMs) of the GLP-1 receptor have been characterized [64,65,66]. These molecules would enhance receptor signaling when the endogenous ligand is present, thereby providing an opportunity to enhance the normal GLP-1 response selectively [64] Some of these PAMS interact with the less active form of GLP-1, potentiating the activity of GLP-1(9−36) by interacting with both the peptide and the receptor [64,65,66].

## 3. Glucagon-like Peptide 1 Receptor Activation

The GLP-1 receptor forms part of the secretin calcitonin and the parathyroid family of G-coupled receptors (GPCRs) [67]. GLP-1 and its receptor agonists can influence a variety of brain functions, including but not limited to satiety, thermogenesis, blood pressure, neurogenesis, neurodegeneration, retinal repair, and altering energy homeostasis [68]. mRNA transcripts of the receptor were widely distributed in peripheral tissues and the central nervous system (CNS) [69]. In this regard, the exact locations of the GLP-1 receptors are still not fully elucidated. However, in rats, GLP-1R mRNA is expressed in pancreatic islets, predominantly in β-cells but also in α and δ-cells, and the lung, stomach, heart, ovary, or kidney [70,71,72]. Using a specific anti-GLP-1R Mab in humans and monkeys, GLP-1R was predominantly localized in β-cells with a markedly weaker expression in acinar cells and in the kidney, lung, heart, and the gastrointestinal tract. The highest GLP-1R expression was in the Brunner’s gland in the duodenum and myenteric plexus neurons throughout the gut. No GLP-1R was seen in primate liver and thyroid [73,74]. GLP-1R mRNA expression is also described in 3T3-L1 preadipocytes and mature adipocytes in mice and humans, or in human epicardial fat and visceral and subcutaneous adipose tissues in various endocrine tumors [32,70,71,72,75,76,77,78,79,80,81,82]. A recent study has demonstrated the GLP-1R expression in peripheral tissues in male mice by a Glp1r.tdTomato reporter mouse expressing the reporter protein, tdTomato, in Glp1r-expressing cells. To complement this, histological mapping, in situ hybridization and GLP-1R immunohistochemistry were performed on the same tissues in the study [83]. With this technique, the authors described known sites and found new, hitherto unknown sites for receptor expression. These included Brunner’s glands, intraepithelial lymphocytes, and myenteric plexus nerve fibers throughout the gastrointestinal tract, as well as some thyroid follicular and parafollicular cells or endothelial cells of the liver, among others [83]. In mice, GLP-1R is present in pancreatic acinar-, β-, and δ cells, but rarely in α cells [83]. This was previously described using Glp1r reporter mice and a validated GLP-1R antibody, in which no expression of the receptor was observed in α-cells, and GLP-1R mRNA but not protein was detected in δ-cells [84]. This demonstrates that there may be differences in GLP-1 effects since GLP-1R expression may vary among species [32,85,86].

In the rodent brain, regardless of the appearance of some differences in receptor expression between rats and mice, and depending on the technique of analysis employed, several studies using chemical biology, recombinant genetics, and super-resolution compatible labeling probes methods have described high content of GLP-1R in the hypothalamus (preoptic area, PVN, supraoptic, arcuate, DMH, lateral and ventromedial nuclei), also in the circumventricular organs such as the AP, and the choroid plexus (CP) [32,87]. Similarly, in non-human primates, the GLP-1R mRNA and protein expression present similar distribution in the CNS to rodents [88]. In the human brain, in situ hybridization histochemistry revealed specific labeling for GLP-1 receptor mRNA in several brain areas like the cerebral cortex (especially in the occipital and frontal cortex), hypothalamus (mainly the ventromedial and arcuate nuclei), hippocampus, thalamus, caudate-putamen, and globus pallidum [89]. 

In the brain, the GLP-1 receptor is highly expressed differently in neurons and glial cells in several regions [24,90,91]. For example, in mice, GLP-1R is expressed in neurons and microglia cells but not in astrocytes in the spinal cord [92]. In the hypothalamus, GLP-1R is expressed in neurons, vascular cells, and in a population of astrocytes that express aquaporin, associated with astrocyte vascular end feet, but not in microglia [93,94]. This observed lack in the expression of the receptor may be related to the fact that it is necessary to trigger an inflammatory response in the brain to increase its density, which indicates that it plays a role in controlling inflammation [22,91].

GLP1-R accumulates in the circumventricular nuclei of the area postrema and median eminence and the adjacent nuclei of the basal hypothalamus and NTS [24,95]. Therefore, astrocytes and tanycytes can play a role in the CNS trafficking of GLP-1 or GLP-1 ligands. Non-acylated, non-PEGylated incretin receptor agonists such as Liraglutide, semaglutide, and Peptide 18 enter the brain [95,96]. However, non-acylated, non-PEGylated incretin receptor agonists such as exendin-4 and DA-JC4 were able to cross the BBB based on their rate of brain influx [96].

Instead, GLP-1, produced by L-cells, binds to the GLP-1 receptor located on afferent neurons within the intestine and portal vein. Acting as a key regulator of the gut-brain axis, it likely influences the brain indirectly through vagal nerve fibers in the enteric area, whereby it transmits metabolic information to the nucleus of the solitary tract (NTS) [68,97].

Moreover, GLP-1R is distributed in the thymus and spleen, and various immune cells such as T cells, natural killer T cells, monocytes, and macrophages can regulate peripheral Treg cell proportion in rodents and humans [98].

It is essential to consider differences in GLP-1R activation in males and females. In this regard, several studies have demonstrated gender differences in the action of GLP-1R agonists. The central administration of exendine-4 increases reward behavior suppression in females, compared to male rats. In the operant conditioning task, in both non-deprived and food-restricted animals with more significant differences in the fed state [99], this gender difference is abrogated with ER antagonist in both sexes [99]. In anxiety-like behavior models, it is described that the expression of GLP-1R in the supramammillary nucleus is sufficient for anxiogenesis in both sexes, but is necessary only in females [52]. Moreover, GLP-1R activation alters performance in cognitive tasks in a sex-dependent manner, since females benefit most from the acute Ex-4 treatment by enhancing the execution of the location discrimination task [100].

Furthermore, in relation to the anti-inflammatory activities of GLP-1 RAs, an apparent sex effect is observed in an animal model of food restriction of the mother. At 21 days of postnatal life, pups show increased hippocampal expression of pro-inflammatory mediators, a number of microglia and astrocytes, and microgliosis only in male rats [101]. The treatment of food-restricted pregnant dams with liraglutide prevents this neuroinflammatory response, promoting the production of anti-inflammatory molecules and decreasing the number and reactivity of microglial cells and astrocytes only in males [101]. With regards to data from preregistration, clinical trials show that liraglutide exposure is about 32% higher in women than in men after adjusting for body weight [102,103]; and different pharmacokinetics have been described with a higher rate of drug degradation in men when compared with women [102,103]. Regarding other parameters, namely HbA1c levels and cardiovascular risk, it remains to be seen whether there is a sex difference. This difference could be partly attributed to the sex-specific pharmacodynamics of GLP-1 RAs, which lead to different drug exposure levels. Alternatively, this dimorphism could be attributed to specific hormonal profiles of the sex [104].

**Figure 2:**
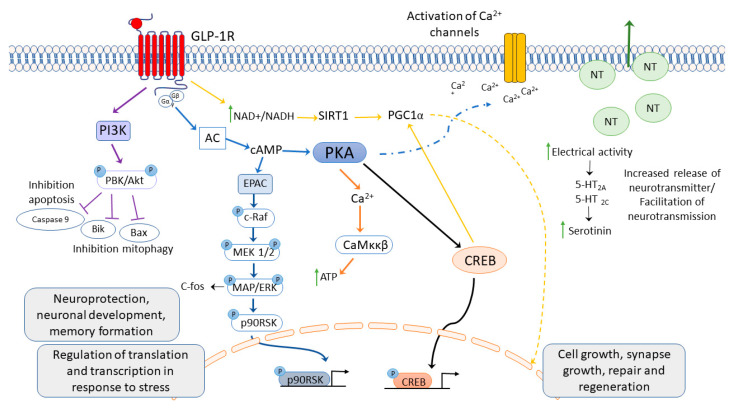
GLP-1 receptor activation induces several second messenger cell signaling pathways.

GLP-1R mediated effects arise because of the immediate signaling cascade, which can quickly impact insulin secretion and calcium flux post-translational modifications. The late stage or chronic effects can operate through modulation of gene expression and cellular metabolism [68]. To induce the neuroprotective effects, the GLP-1R must be activated in the brain (Figure 2). Upon agonist binding, the GPCR undergoes a conformational change and transmits extracellular signals through heterotrimeric G-proteins, which consist of Gα and Gβγ subunits [105]. The GLP-1 initiates a cascade that involves the activation of membrane-bound adenyl cyclase (AC) and the consequent production of cyclic adenosine monophosphate (cAMP). Downstream of cAMP formation, several signal transduction pathways can be initiated, which generally require activation of either one or both of the cellular cAMP effectors, protein kinase A (PKA), and exchange protein directly activated by cAMP (EPAC) [25,68]. However, different GLP-1R ligands can engage selective pathways to elicit a different cellular response [106].

Activation of GLP-1R induces cellular response to neuroprotection, neurodevelopment, memory formation, cell growth, synapse growth, repair, and regeneration, increases neurotransmitters’ release, and activates Ca^2+^ channels. Abbreviatures: PI3 K: phosphoinositide 3 kinase; PKB: protein kinase B; AC: adenylate cyclase; EPAC: exchange proteins directly activated by cAMP; MAPK: mitogen-activated protein kinase; ERK: extracellular signal-regulated kinase; CREB: cyclic AMP response element-binding protein; P90RSK: ribosomal S6 kinase; MEK1/2: MAPK or Erk kinases; c-Raf: cellular Raf gene (rapidly accelerated fibrosarcoma); Casp-3: caspase 3; Bax, Bik: Bcl2-interacting killer; Ca^2+^: calcium ions; SIRT1: sirtuin 1; PGC1α: peroxisome proliferator-activated receptor γ co-activator 1 α. Modified from [36,68].

## 4. Anti-Inflammatory Effects of GLP-1R Activation in Neurodegenerative Diseases

GLP-1 is a target and mediator of the inflammatory response [56]. Inflammatory stimuli can increase GLP-1 secretion, and GLP-1, in turn, modulates inflammation in multiple sites [56]. Experimental inflammation increases circulant GLP-1 levels in rodents [107,108]. Furthermore, GLP-1 shows anti-inflammatory effects on pancreatic islets and adipose tissue [109,110], also in the liver, the vascular system, including aorta and vein endothelial cells, kidney, lung, testis, and skin, by reducing the production of inflammatory cytokines and infiltration of immune cells in the tissues [23,31,56,111]. Therefore, GLP-1 emerges as a therapeutic target for treating inflammatory diseases in the brain.

### 4.1. Multiple Sclerosis

Multiple sclerosis (MS) is the most prevalent chronic inflammatory disease of the central nervous system (CNS) and is currently incurable [112]. It is punctuated by fully or partially reversible episodes of neurological disability, usually lasting days to weeks [113]. Typical MS manifestations include visual, sensory, motor, and sphincter disturbances, incoordination, gait disorder, and cognitive impairment [113,114]. The variation in clinical manifestations correlates with the spatiotemporal dissemination of lesioned sites of pathology within the CNS [112]. These lesions are a hallmark of MS and are caused by immune cell infiltration across the blood-brain barrier (BBB) that promotes inflammation, demyelination, gliosis, and neuroaxonal degeneration, leading to disruption of neuronal signaling [112]. MS lesions can appear throughout the CNS and are most easily recognized in the white matter as focal areas of demyelination, inflammation, and glial reaction [113].

GLP-1R agonists have been reported to have therapeutic efficacy on several MS animal models and in “in vitro” models [115,116,117,118]. The experimental autoimmune encephalitis (EAE) is a classical animal model mimicking central nervous demyelinating lesions and T-cell responses of MS [119]. Pictures acute, chronic relapsing, acquired, inflammatory demyelinating autoimmune disease [120]. The cuprizone (Cup) model in mice induces highly reproducible demyelination of distinct brain regions, among them the corpus callosum (CC) [121]. For the studies in vitro, the BV2 cell line (immortalized microglia cells) is used [122].

It has been determined in EAE mice that the immunization with MOG affects the expression of GLP-1R [121]. In the lymph node or the spinal cord, at the early stage of EAE when priming of the immune response occurs, no changes occur in the GLP-1R expression at PID 8 measured by Western blot [121]. However, mRNA expression levels tested by RT-PCR are downregulated after EAE induction in the spinal cord [115]. Furthermore, a decrease in GLP-1R was described in the spinal cord of sick EAE mice at the late stage of EAE (PID 42) [121,123]. In the brain, no alteration in the GLP-1R expression in the early or late stages of EAE was observed in mice [121]. 

The treatment with Liraglutide, exendin-4, or dulaglutide ameliorates the disease score in EAE and Cup-mice [115,116,117,121,123,124] independently of the dose, time, and via administration (subcutaneously or intraperitoneally). The treatment with NLY01 at the onset of disease in relapsing-remitting EAE in SJL mice decreases the EAE clinical score and second relapse [121]. These compounds delay the onset and reduce the severity in cuprizone-treated mice and EAE mice and rats [115,116,117,121,123,124]. Furthermore, Liraglutide can ameliorate cuprizone-induced behavioral abnormalities and improve myelinization in the corpus callosum in these mice or in the spinal cord of EAE mice [115,116]. Demyelination is a key feature of multiple sclerosis neuropathology [112]. Loss of different myelin proteins, such as myelin-associated glycoprotein (MAG), myelin basic protein (MBP), myelin oligodendrocyte protein (MOG), proteo-lipid protein (PLP), or CNPase occurs to the same extent [120]. Cup administration reduces the MBP expression and myelin sheath loss, an effect that is reverted by liraglutide administration in the corpus callosum [116]. Liraglutide treatment also elevates the percentage of myelinated nerve fibers in this brain region, and restores the oligodendrocyte progenitor cells’ (OPCs) re-myelination ability via Olig2 transcription activation [116].

Related to the anti-inflammatory effects of GLP-1R activation, NLY01 suppresses the activation and expansion of innate immune cells in the myeloid lineage at the early stage of EAE. This is demonstrated by the reduction of spleen weight and the absolute number of CD45^+^ splenocytes induced by the immunization [121]. This compound induces a reduction in the percentage and absolute counts of CD11b^+^ cells, including neutrophils (CD11b^+^Ly6G^+^ cells), activated myeloid cells (CD11b^+^MHCII^+^), and dendritic cells (CD11b^+^CD11c^+^) [121]. At the pre-onset stage, PID 11, NLY01 reduces the percentage of activated myeloid cells (CD11b^+^MHCII^+^), mature dendritic cells (CD11c^+^MHCII^+^), and effector T cells (CD4^+^CD44^+^) in the spleen [121]. Without directly affecting activation, proliferation, and differentiation of T cells into Th1 or Treg [121], as was observed with dulaglutide, that does not change the percentage of Treg cells in the CNS of EAE mice [117]. However, dulaglutide reduces the number of macrophages (both M1 and M2) in the CNS [117]. GLP-1RAS may prevent the activation of myeloid cells in the periphery at the early stage of EAE [117,121]. Mice that lack a stable Th17 population and are deficient for RAR-related orphan receptor gamma (RORγt), IL-6, IL-23 p19, and IL-23R in mice are resistant to EAE development, showing the importance of Th17 cells for developing EAE [119]. In the spleen of EAE mice, the mRNA expression of RORγt and Th1 cell-related mRNA T-box 21 (Tbet) related to Th17 cells are increased [115], and the treatment with Liraglutide can reduce its mRNA expression [115]. In addition, the expansion of circulating neutrophils, a hallmark of EAE [125], is reverted by NLY01 [121]. 

Infiltration of immune cells from the periphery has been the main target of currently available therapies for multiple sclerosis [112]. It is established in the CNS extrinsic (peripheral) model that autoreactive T cells, activated at peripheral sites, traffic to the CNS along with activated B cells and monocytes [112]. The EAE model generates pathogenic CD4^+^ T helper 1 (TH1) cells and TH17 cells in the draining lymph nodes. These cells then enter the circulation and ultimately exert their effector functions within the CNS, crossing the BBB or the blood-cerebrospinal fluid (CSF) barrier at the choroid plexus [112]. CNS-intrinsic events may trigger disease development, with the infiltration of autoreactive lymphocytes as a secondary phenomenon [112]. It has been described that dulaglutide treatment reduces lymphocyte infiltration into the CNS at PID 14 in EAE-mice [117]. Also, dulaglutide suppresses the development of highly encephalitogenic Th1/Th17 cells in the CNS, suggesting a critical role in the modulation of T cell pathogenicity in the CNS [117].

Furthermore, NLY01 lowers the percentage of infiltrating leukocytes (CD45^high^ cells) and effector/memory T cells (CD4^+^CD44^+^) in the CNS [121]. Moreover, this novel GLP-1RAS decreases Clec12a^+^ cells in EAE mice, essential for migrating myeloid cells into the CNS tissues during inflammation [121]. It also reduces the expression of chemokines (Cc18 and Cxc11) in the hindbrain of EAE mice [121]. 

In MS, the excessive immune response is a feature of disease progression in which the production of diverse proinflammatory cytokines is a well-known pathogenic event [126,127]. It results in the production of several interleukins (IL) such as IL-1, IL-6, IL-12, IL-18, and IL-23 that promote the induction and expansion of Th1 and Th17 cells [128]. Following the breakdown of BBB, activation of Th1, Th17, and γδ T cells enter the brain and spinal cord [128]. In addition, resident microglia of the CNS, infiltrating monocytes, and neutrophils secrete IL-1β and IL-23 to activate further and expand these cells [128]. Exendin-4 administration decreased the mRNA expression level of proinflammatory cytokines in the spinal cords of EAE mice. Similarly, in LPS-stimulated microglia, exendin-4 exposure reduced the mRNA expression of IL-1β and TNF-α, but not IL-6 [123].

In EAE, inflammation and activation of the inflammasome are initiated by the binding of pathogen-associated molecular patterns (PAMP) or danger-associated molecular patterns (DAMP), like the high mobility group box protein-1 (HMGBP) that is released to the extracellular environment from the activated macrophages, monocytes, dendritic cells as well as apoptotic oligodendrocytes (highly expressed in relapsing-remitting MS (RRMS) patients) [128,129,130]. These molecules bind to PPR such as Toll-like receptors (TLRs) [99]. Activation of TLR recruits the adaptor protein myeloid differentiation primary response 88 (MYD88) into the receptor complex, leading to phosphorylation of the inhibitor of nuclear factor-kB (NFκB) IκB through interaction with the p50 and p65 transcription factors. Activation of the NF-κB pathway causes the synthesis of pro-IL-1-β or pro-IL-18 [128]. Exendin-4 treatment attenuates NF-κB p65 expression in the EAE spinal cord and blocks IκBα degradation in LPS-stimulated primary microglia, further demonstrating that exendin-4 can suppress microglial polarization [123]. 

Moreover, inflammasome activation plays a critical role in the autoimmune and proinflammatory responses in MS [128]. Inflammasome assembly results in caspase-1 activation that leads to the processing and release of IL-1b and IL-18 [128,129]. It compromises the BBB, induces neural toxicity and stimulates autoimmune T cells, and then deteriorates MS/EAE [129,130]. Moreover, NLRP3 inflammasome activation through active caspase-1 cleaves gasdermin D (GSDMD), that initiates pyroptosis leading to upregulation of IL-1β [128]. EAE increases the mRNA expression level of NLRP3, ASC, caspase 1, and GSDMD [129], and the treatment with liraglutide downregulates their expression [115]. Liraglutide also prevents Cup-induced overexpression of HMGB1 and TLR-4 indirectly and directly, respectively [116]. It attenuates the activation and release of the inflammatory cytokines such as caspase-1 and IL-1β via mitigating the Cup-induced NLRP3 overexpression [116].

Furthermore, another way to activate the neuroprotective anti-inflammatory downstream signaling cascade is through the AMPK system [131]. GLP-1 R activation induces AMPK phosphorylation, and activates multiple downstream pathways through molecules such as SIRT1, p53, and peroxisome proliferator-activated receptor γ coactivator-1 (PGC1α) [131,132]. This then inhibits the nuclear factor-kB (NFκB) and indirectly suppresses proinflammatory gene transcriptions [132,133]. In EAE and cup MS models, the lumbar spinal cord and brain exhibit reduced phosphorylated AMPK expression, and liraglutide administration partly restored the expression levels [115,116].

In the EAE spinal cord, the number of amoeboid Iba1-positive microglia is increased, and exendin-4 affects their morphological transformation and reverse into ramified cells [123]. It is described that activated microglia induces A1 astrocytes by secreting Il-1α, TNF, and C1q, and these cytokines together are necessary and sufficient to induce A1 astrocytes. A1 astrocytes lose the ability to promote neuronal survival, outgrowth, synaptogenesis, and phagocytosis and induces the death of neurons and oligodendrocytes [134]. In this regard, it has been described that NLY01 interferes with the glial activation cascade, blocking the formation of neurotoxic astrocytes and preventing retinal degeneration and RGC loss in EAE mice [121].

### 4.2. Amyotrophic Lateral Sclerosis (ALS)

Amyotrophic lateral sclerosis (ALS) is a devastating neurodegenerative disease characterized by selective degeneration of upper and lower motor neurons, leading to muscle atrophy, paralysis, and death [135]. ALS is a remarkably progressive disease, with death occurring typically 3–5 years from symptoms onset, usually due to respiratory failure [135]. No effective treatments for the disease are currently available, mainly due to the high degree of complexity and heterogeneity that characterizes the disease [135]. Two ALS types, familial ALS (fALS) and sporadic ALS (sALS) are frequently described [136]. In fALS, Cu/Zn superoxide dismutase-1 mutations have been described in the superoxide dismutase 1 (SOD1) gene, but 40 genes should be related to ALS [137]. Pathogenic processes lead to ALS neurodegeneration, including mitochondrial dysfunction, apoptosis, chronic inflammatory reactions mediated by cytokine production, free radical generation, decreased neurogenesis, synaptic loss, synaptic death, excitotoxicity, axonal transport impairment, and neurotrophic factor dysregulation [137].

In the spinal cords of the SOD1 mouse model of ALS, microglia activation occurs robustly, as well as in ALS patient tissue and mutant SOD1 transgenic mice [138]. In in vitro studies, it has been described that ALS microglia is proinflammatory and neurotoxic [139]. These microglia upregulate factors that have been linked to neurotoxicity, including Nox2 [140] and pathways of Alzheimer’s Disease (AD) during the disease progression (MAPT (tau), PSEN2, and APOE genes) whose mutations are directly linked to familial AD [140]. ALS microglia may be a double-edged sword, with SOD1^G93A^-induced negative changes being counterbalanced by a neuroprotective response mediated by extrinsic regulatory factors, including signals released by dying motor neurons and infiltrating T cells. These microglia can induce factors that can suppress neurodegeneration, including IGF-1 (Igf1) and progranulin (Grn), which have been shown to mediate motor neuron protection in ALS animal models [140,141,142].

Astrocytes from fALS and sALS patients are heterogeneous and express 22 upregulated genes encompassing chemokines, proinflammatory cytokines, and components of the complement cascade, many of which are implicated in ALS [136,143]. A network-based pathway analysis using Ingenuity Pathway Analysis software has identified different networks: the NF-κB signaling complex is identified as a significant interactor, and IFN-α is also identified within this gene network interactome. The second-ranked network identifies pathways involving kinase signaling such as MAPK, JNK, and AKT, which establish many interactions with the inflammatory genes in this cluster [136].

Recently, increased plasma levels of GLP-1 have been identified in ASL patients, other metabolic biomarkers and adipokines [144]. However, in Transgenic (Tg) TDP43^A315T^ mice, an animal model of ALS, no changes were observed in circulating GLP-1 at onset or the end-stage of the disease [145]. Regarding the use of GLP-1 RAs as a possible therapeutical target, there are currently few studies investigating the role of GLP-1 receptor activation in treating ALS. 

Purified embryonic motor neurons derived from non-transgenic and mutant SOD1-G93A transgenic mice show neuroprotective effects of the GLP-1 analog, N-acetyl-GLP-1(7–34) amide against excitotoxic damage [146]. In addition, in cell culture (NSC-19 neuroblastoma cells) exendin-4 proved to be neurotrophic. This elevated choline acetyltransferase (ChAT) activity, as well as neuroprotective protecting cells from hydrogen peroxide-induced oxidative stress and staurosporine-induced apoptosis [147].

The effects of an injection of GLP-1 releasing mesenchymal stromal cells (MSCs) into the brain of mutant transgenic SOD1G93A mice have been analyzed [148]. The lifespan is extended, the motor neuron impairment is delayed, and the chronic response in the CNS is reduced [148]. Furthermore, the administration of Exendin-4 to SOD1 G93A mutant mice via a subcutaneous osmotic pump provides a steady-state infusion, improves glucose tolerance, normalizes running wheel behavior, and attenuates neuronal cell death in the lumbar spinal cord [147]. However, using two strains of transgenic mice that replicate features of ALS, an optimized SOD1G93A transgenic model and a TDP-43Q331K transgenic line, liraglutide administration fails to slow the disease progression of ASL, and does not have any effect on motor neuron counts or glial activation in lumbar spinal cords [149].

### 4.3. Alzheimer’s Disease (AD)

Alzheimer’s Disease (AD) is the most common cause of dementia [150]. It is a neurodegenerative disease that causes an amnestic cognitive impairment in its prototypical presentation and non-amnestic cognitive impairment in its less common variant [150]. AD is biologically defined by the presence of β-amyloid-containing plaques and tau-containing neurofibrillary tangles [150]. The neurotoxicity of Aβ requires self-assembly of the 4 kDa Aβ peptide into aggregates of various sizes that are deposited as amyloid plaques and that are easily detected in AD brains [151]. However, a self-association of Aβ and Aβ self-aggregates exists to form neurotoxic-soluble oligomers that have been observed at the postsynapse in AD hippocampi, with elevated levels in the brain and cerebrospinal fluid of AD patients [151]. The best correlate of the extent of dementia is not an amyloid burden but rather synapse loss [152,153]. It is hypothesized that synapse failure and neuronal dysfunction derive from the impact of Aβ oligomer that is intimately related to defective insulin signaling [151,152,153,154].

Moreover, damaged neurons and neurites in the AD brain, highly insoluble amyloid β peptide deposits, and neurofibrillary tangles provide potent stimuli for inflammation [155]. These stimuli are discrete, micro localized, and present from early preclinical to terminal stages of AD [155]. Local upregulation of complement, cytokines, acute phase reactants, and other inflammatory mediators is also discrete, micro localized, and chronic [155]. Brain inflammation orchestrated by glial cells plays a crucial role in synapse damage/ elimination and brain dysfunction, leading to cognitive and non-cognitive symptoms of AD [156]. Increased expression and release of proinflammatory cytokines (TNF-α, IL-1β, and IL-6) have been described both in vitro and in vivo [156]. However, considerable evidence indicates that pathological alterations occur in central and peripheral immune responses and change over time [157].

Epidemiological studies found a correlation between type 2 diabetes mellitus (T2DM) and an increased risk of developing AD or other neurodegenerative disorders at a later stage [158]. Moreover, several pathological features, including impaired insulin signaling and inflammation, appear to be shared by diabetic and AD patients [152]. Furthermore, in neurons exposed to amyloid-β oligomers, the insulin receptors are removed from the plasma membrane, suggesting that a state of neuronal insulin resistance occurs in AD [151,156]. In this context, GLP-1 RAs emerge as the target for therapeutical treatment for AD. In established animal models of Alzheimer’s disease, the APP/PS1 mice (a transgenic mouse model of AD), expresses the human Swedish mutated form of APP and a mutated human form of PS-1 [158]. The AD/streptozotocin (STZ) model GLP-1 analogs show neuroprotective and anti-inflammatory effects [36].

Neuroinflammation associated with microglia and astrocyte interaction, and increased reactive astrocytes population, has been seen in AD [159]. The death of neurons in AD is partly dependent upon reactive astrocytes through Aβ-activated microglia [159]. The selective blockade of Aβ-induced activation of GLP-1R^+^ microglia by subcutaneous administration of NLY01 prevents reactive astrocytes conversion, neurodegeneration, and cognitive deficits without toxicity in animal models of AD [159]. Targeting GLP-1R expressed on microglia by NLY01 selectively inhibits microglial activation and induction of the reactive astrocyte inducers TNF-α, C1q, and IL-1α, thus protecting both mouse (5xFAD and 3xTg-AD mice) and human neurons in vitro (human embryonic stem cell (hESC)-derived human cortical neurons) [159]. Moreover, in 5xFAD mice, exenatide reduces Aβ deposition, the expression of NLRP2 inflammasome in astrocytes, and TNF-α and IL-1β expression in the piriform cortex [160].

Liraglutide reduces by 40–50% the overall β-amyloid plaque count and dense-core plaque numbers, and by 50% the number of activated microglia in the cortex [161] and the hippocampus [162] of APP/PS1 mice. Liraglutide also exerts neuroprotective effects and reduces cortical microglia burden and number in the proximity of amyloid plaques in APP/PS1x*db*/*db* and APPswe/PS1dE9 mice [163,164]. It also reduces astrocytosis in APPswe/PS1dE9 mice [164]. Besides, in APP/PS1 mice, lasting liraglutide treatment (8 weeks/i.p/once daily) to mice starting at 8 weeks of age reduces by 47% the microglia staining marker, the ionized calcium-binding adaptor molecule 1 (Iba1) in the hippocampus, showing a prophylactic effect [165].

Using different experimental models of AD, ranging from hippocampal cell cultures to mice to non-human primates (NHPs), Batista et al. describe how liraglutide prevents the loss of brain insulin receptors and synapses by activation of the PKA signaling pathway [166]. Systemic treatment of NHPs with liraglutide provides partial protection by decreasing AD-related insulin receptors, synaptic, and tau pathology in specific brain regions [166].

In APP/PS1 mice, the GLP-1/GIP dual receptor agonists DA4-JC or DA-JC1, specific for those receptors have apparent neuroprotective effects [167,168]. Compared to liraglutide, the dual receptor agonist is superior in most parameters tested, showing anti-inflammatory effects [167,168] by decreasing TNF-α and IL-1β production, and lowering the amyloid plaque load [167]. In the triple APP/PS1/tau mouse AD model, DA4-JC improves cognition and downregulates amyloid or p-tau, among many other neuroprotective effects [169]. Moreover, AD/STZ mice DA4-JC reduces microglia and astrocyte activation [170].

The triple receptor agonist (TA; GLP-1/GIP/glucagon) reduces the reactivity of microglia and astrocytes in APP/PS1 mice, and reduces the total amount of β-amyloid and oxidative stress in the cortex and hippocampus [171].

In this context, several drugs used to treat T2DM and normalize insulin signaling are being tested in clinical trials for AD treatment [172,173].

### 4.4. Parkinson’s Disease (AD)

The pathological hallmarks of Parkinson’s disease (PD) are a loss of dopaminergic (DA) neurons in the substantia nigra pars compacta (SN), the presence of proteinaceous inclusions termed Lewy bodies and neurites [174], abnormal deposition of α-synuclein (α-syn), mitochondrial dysfunction, disruption of the homeostasis between autophagy and apoptosis, and motor impairments such as rest tremor, rigidity, and bradykinesia [175,176]. Among neurodegenerative disorders, PD is the second most frequent and fastest-growing neurological disease [177]. In recent years, the relationship between T2DM and PD has been demonstrated; T2DM contributes to disease onset and modifies motor and nonmotor symptoms [177,178]. Also, recent studies suggest intestinal inflammation may contribute to the development of neurodegenerative conditions. Individuals with Parkinson’s exhibit inflammation and oxidative stress in the gut characterized by constipation, intestinal permeability, dysbiosis, and increased levels of potentially pathogenic forms of enteric αSYN [131,179].

Chronic inflammation is a crucial aspect of PD. The activation of microglia and astrocytes lead to the accumulation of cytokines, NF-κB pathway activation, and oxidative damage to proteins. This was described in the CSF and brains of individuals with PD, which is also evident in post-mortem PD brains at autopsy [174,180]. Inflammation-derived oxidative stress and cytokine-dependent toxicity may contribute to nigrostriatal pathway degeneration and hasten disease progression in humans with idiopathic PD [180].

Many studies have demonstrated the neuroprotective effects of GLP-1 R stimulation in PD models, improving motor and non-motor deficits [177,181]. Exendin-4 was the first GLP-1 RAS to have effects in preclinical tests in PD patients [182]. Exendin-4 and DA-CH5 protect against 6-hydroxydopamine (6-OHDA) cytotoxicity in SH-SY5Y cells by inhibiting apoptosis, improving mitogenesis, and inducing autophagy flux [183].

It has been shown that liraglutide, semaglutide, exendin-4, NLY01, and the GLP-1/GIP dual receptors agonist (DA3-CH, DA5-CH, DA-JC4, and DA-JC1), have neuroprotective properties in the in 1-methyl-4-phenyl-1,2,3,6-tetrahydropypridine (MPTP) mouse model [184,185,186,187,188,189,190,191].

Related to the anti-inflammatory role of GLP-1 RAs, it has been demonstrated that liraglutide, exendin-4, DA3-CH, DA5-CH, and DA-JC1 reduce the activation of microglia and astrocytes in the MPTP mouse model of PD [184,185,186,187,191]. DA5-CH reduces inflammation markers such as TLR4, Iba-1, GFAP, NF-κB, TNF-α, and transforms the growth factor β1 (TGF-β1) in MPTP mice, as well as the proinflammatory cytokines (IL-6 and IL-Iβ) [187]. In 6-OHDA-induced rat PD, exendin -4 and DA-CH5 reduce the activation of astrocytes and the expression of IL-1β and TNF-α in the striatum [183].

The mouse strain with the constructed commensal MG1363-pMG36e-GLP-1 was engineered to continuously express GLP-1 to treat Parkinson’s disease in an MPTP-treated Parkinson’s disease model [192]. MG1363-pMG36e-GLP-1 is beneficial for relieving inflammation in PD mice since it reduces TLR-4 expression, down-regulates p-NF-κB in the NF-κB signaling pathway and reduces the expression of proinflammatory factors IL-1β, IL-6, and TNF-α at both the gene and protein levels. Although, some of the beneficial effects of the constructed commensal MG1363- pMG36e-GLP-1 in this PD mouse could be independent of GLP-1 production and mediated by the commensal itself through another mechanism [192] 

As mentioned previously, AMPK plays a role in inflammation signaling for multiple sclerosis. AMPK activation indirectly inhibits NF-kB [131]. Liraglutide increases p-AMPK expression in MPTP mice and reduces NF-κB protein levels [185]. The AMPK pathway signaling is not described yet in this animal model under GLP-1 RAS treatment. Also, other roles of AMPK on dopaminergic neurons are contradictory. It is not yet possible to conclude whether the elevation in pAMPK levels causes harm to dopaminergic neurons or can promote neuronal survival [131].

Using a single-blind trial design, a proof of concept has evaluated the progress of 45 patients with moderate Parkinson’s disease (PD), randomly assigned to receive subcutaneous exenatide injection for 12 months or as controls [193]. Preliminary clinical trials show that exenatide-treated patients have a mean improvement at 12 months on the MDS-UPDRS of 2.7 points, compared with a mean decline of 2.2 points in control patients [193]. There are four clinical trials currently ongoing for testing semaglutide (NCT03659682), exenatide (NCT04305002, NCT03456687), and Liraglutide (NCT02953665) in PD.

## 5. Discussion and Concluding Remarks

The inflammatory response is coordinated by an extensive range of mediators that form complex regulatory networks [194]. Despite this complexity, all inflammatory responses can be divided into four common components of the inflammatory pathway: inflammatory inducers, sensors, mediators, and target tissues [194,195].

In the neurodegenerative diseases studied in this review, the inducers of the inflammation differ. However, the sensors and mediators are similar, produced by resident CNS glia (microglia and astrocytes), endothelial cells, and peripherally derived immune cells [179], causing different brain alterations. The maintained activation of CNS glia (microglia and astrocytes) with significant cytokine and chemokine production, infiltration of peripheral immune cells, edema, increased blood-brain barrier (BBB) permeability, and breakdown are related to pathological neuroinflammation [11]. It is also related to immune, physiological, biochemical, and psychological consequences [11]. However, the degree of neuroinflammation depends on the context, duration, and course of the primary stimulus or insult [11].

In MS, peripherally activated T cells cross the blood-brain barrier (BBB) into the CNS, where they are re-activated and secrete cytokines to exert their effector functions [196]. This cytokine secretion leads to the activation of CNS-resident immune cells (such as microglia, astrocytes, and macrophages), as well as to the production of cytokines, increased antigen-presenting cell (APC) function, and the enhanced production of reactive oxygen species (ROS) and reactive nitrogen species [196]. CD8 T cells can also release cytolytic granules causing axonal dissection [196].

In ALS, a genetic predisposition is described [137]. However, developmental factors such as maternal age, childhood infections, and environmental risk factors are also related to ALS development [197]. The mechanisms underlying neurodegeneration in ALS are multifactorial and operate through inter-related molecular and genetic pathways [197]. Neurodegeneration in ALS might result from a complex interaction of glutamate excitotoxicity, generation of free radicals, cytoplasmic protein aggregates, SOD1 enzymes, combined with mitochondrial dysfunction, and disruption of axonal transport processes through the accumulation of neurofilament intracellular aggregates. Mutations in TARDBP and FUS result in intracellular aggregates that harm neurons. Activation of microglia results in the secretion of proinflammatory cytokines, resulting in further toxicity. Ultimately, motor neuron degeneration occurs by activating calcium-dependent enzymatic pathways [197].

In AD, genetic contributions represent only a modest part of the attributable risk [150]. Several potentially modifiable risk factors in midlife, particularly metabolic factors (diabetes mellitus, hypertension, obesity, and low HDL cholesterol), hearing loss, traumatic brain injury, and alcohol abuse, are associated with an increased risk of later-life dementia [150]. The pathology of canonical AD dementia involves Aβ-containing extracellular neuritic plaques in a widespread distribution throughout the cerebral cortex [150]. The impact of Aβ oligomers in the brain appears intimately related to defective insulin signaling [152]. Other proteins important for synaptic plasticity, including NMDA-and AMPA-type glutamate receptors, are removed from the cell surface when neurons are exposed to oligomers, indicating a broad impact on synapses [152]. As the disease progresses, Aβ deposits, neurofibrillary tangles, damaged neurons, brain insulin resistance, and ER stress are thought to provide feedback stimuli for inflammation [151,152]. In this regard, microglial activation and inflammation-mediated neurotoxicity are suggested to be important in the pathogenesis of AD [152].

In PD, the underlying pathological is an injury to the dopaminergic projections from the substantia nigra pars compacta to the caudate nucleus and putamen (striatum) [175]. Intraneuronal Lewy bodies and Lewy neurites are the pathological hallmarks of the disease [175]. Neurodegeneration could be related to mitochondrial dysfunction, oxidative stress, excitotoxicity, apoptosis, and inflammation, in which microglia play a key role [174,175].

All neurological diseases possess some inflammatory component, and microglia are important contributors to brain pathology [198]. Microglia processes monitor the release of ATP, a main attractant and stimulus of microglia, the entry of pathogens and fibrinogen, synaptic function, and activation of neurons. Microglia’s rapid and reversible responses to environmental changes are partly possible by the activation of ion channels and cell surface receptors [198,199]. During many diseases, microglia lose their homeostatic molecular signature and functions, such as their roles in synaptic plasticity, and become chronically inflammatory [200]. Notably, a common neurodegenerative microglial signature has been identified for diseases such as ALS, AD, and MS [200]. Microglia react to injury through morphological changes, increased proliferation, migration to the target, phagocytosis, activation of the NLRP3 inflammasome, and consequently, the release of proinflammatory mediators [198,201].

**Figure 3:**
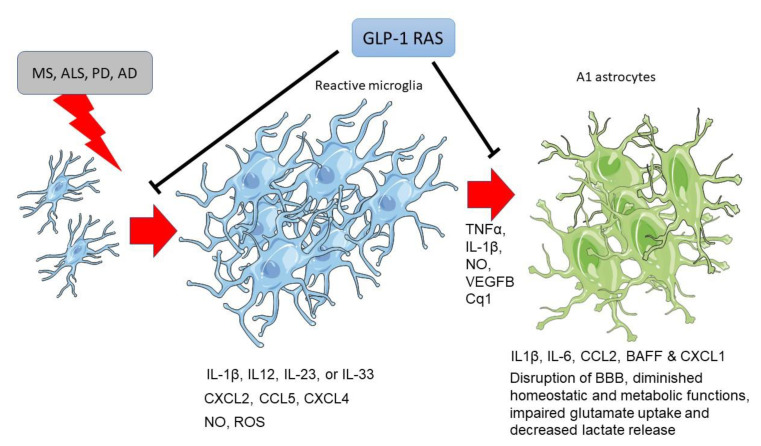
Microgliosis and astrocytosis induced by neurodegenerative diseases are blocked by GLP-1 receptor activation. Abbreviatures: IL-1β: interleukin-1β; IL-12: interleukin-12; IL-23: interleukin-23; IL-33: interleukin-33; IL-6: interleukin-6; CXCL2: Chemokine (C-X-C motif) ligand 2; CCL5: CC chemokine ligand 5; CXCL4: chemokine (C-X-C motif) ligand 4; CCL2: C-C Motif Chemokine Ligand 2; BAFF: B cell–activating factor of the TNF family; CXCL-1: chemokine (C-X-C motif) ligand 1; TNF-α: Tumor necrosis factor α; NO: nitric oxide; VEGFB: Vascular Endothelial Growth Factor B; ROS: Reactive oxygen species; Cq1: complement component 1q.

Microglia are activated by phenotypical morphological changes in response to injury or disease, increasing cell proliferation (Figure 3) [6,11]. GLP-1 R activation decreases the number and reactivity of microglia in MS, ASL, AD and PD [121,123,147,167,168,170,182,185,186,191]. Microglia are the first cells to respond to CNS insults, followed by reactive astrocytosis [15]. Astrocytes’ activation may be either beneficial, promoting tissue repair and homeostasis, or detrimental, exacerbating inflammatory reactions and tissue damage, depending on the stimuli’ nature [15]. At this step, microglial secreted molecules: TNFα, IL-1β, NO, VEGFB, or complement factor Cq1, induce A1 reactive astrocyte phenotype [134,202,203], which is associated with diminished homeostatic and metabolic functions, impaired glutamate uptake (resulting in excitotoxicity) and decreased lactate release from astrocytes [15,134]. GLP-1 R activation (Figure 3) can decrease astrocytosis induced by microglia in these neurodegenerative diseases [121,159,183,184,185,186,187,191]. Moreover, GLP-1 RAS decrease the trafficking of immune cells from the periphery [117,121], a key target of current therapies for multiple sclerosis [112].

**Figure 4:**
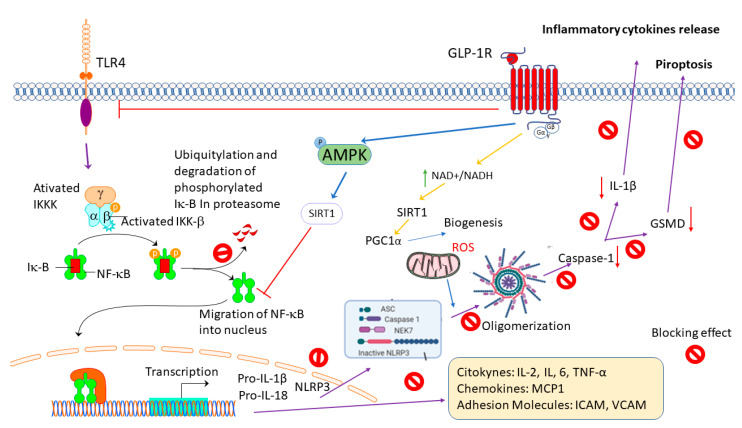
GLP-1 receptor activation induces an anti-inflammatory response. Abbreviatures: NFκB: Nuclear Factor kappa B; IKKK: IκB kinase kinase; IKKβ: IκB kinase β; pro-IL-1β: pro-interleukin-1β; pro-IL-18: pro-interleukin-18; ASC: Apoptosis-associated Speck-like protein containing a CARD; NEK7: NIMA Related Kinase 7; AMPK: adenosine monophosphate-activated protein kinase; SIRT1: Sirtuin 1; GSMD: Gasdermin D; IL-1β: interleukin-1β; IL-2: interleukin-2; IL-6: interleukin-6; MCP1: Monocyte chemoattractant protein 1; ICAM: Intracellular adhesion molecule-1; VCAM: Vascular cell adhesion molecule-1; NAD/NADH: Nicotinamide adenine dinucleotide. Partially Biorender. com used.

GLP-1 RAS emerge as potent anti-inflammatory molecules that are able to modulate glial activation and cytokine production in several pathologies and conditions in the brain [36]. Activation of the GLP-1 receptor (Figure 4) may directly or indirectly inhibit NKκB transcription through the attenuation of NF-κB p65 expression and the blocking of IκBα degradation [123,187]. Alternatively, the DAMPs, like HMGBP, are released to the extracellular environment from the activated macrophages, monocytes, dendritic cells, and apoptotic oligodendrocytes bind to the TLR, attenuating NKκB translocation and the proinflammatory mediators’ production [116,192]. Moreover, blocking NKκB transcription may prevent the production of pro-IL1β and NLRP3 oligomerization [129,167,168]. Therefore, the activation of caspase 1 that produces IL-β cleaves GSDMD-inducing proptosis and releasing proinflammatory cytokines [115,167,168]. In addition, activating the neuroprotective anti-inflammatory downstream signaling cascade through the AMPK system may inhibit nuclear NFκB [131]. The phosphorylation on AMPK can activate SIRT-1 or PGC1α, inhibit NFκB, and indirectly suppress proinflammatory gene transcriptions blocking the synthesis of proinflammatory mediators (cytokines, chemokines, and adhesion molecules) [116,129,185].

## 6. The Limitation of the Study

The incidence and prevalence of Parkinson’s disease are more significant in men than in women [204,205]. In contrast, women have a higher prevalence and incidence of Alzheimer’s disease [205,206], a higher incidence of multiple sclerosis, and a higher relapse frequency than men, although the course of the disease is more benign in women [205,207]. Studies have shown that glia reacts to pathological insults with sex-specific neuroprotective and regenerative effects. At least three factors determine this sex-specific response of glia: sex chromosome genes, gonadal hormones, and neuroactive steroid hormone metabolites [205]. Thus, the incidence, prevalence, age of onset, pathophysiology, and symptomatology of the illness, or even in some cases, its response to therapeutic interventions, may be different in males and females [205].

Regarding GLP-1 RAS, many pharmacokinetic and pharmacodynamic factors are potentially responsible for relevant gender-related differences in their effects [102]. In this regard, most preclinical, in vitro, or ex vivo studies are performed only in males; even sex is not described. In addition, primary culture cells are extracted from males or mixed cells from males and females. Most preclinical studies are not designed to find gender differences. There is still much to be learned regarding the specific molecular and pathogenic cellular mechanisms that determine sex-specific characteristics and the mechanisms of the GLP-1R activation.

## 7. Conclusions

GLP-1 and its long-lasting recombinant analogs display a broad range of neuroprotective and anti-inflammatory effects in several animal models of neurodegenerative diseases. Furthermore, the preclinical results are encouragingly translated to the clinic, since these molecules demonstrate positive results in clinical trials in AD and PD patients. Better mechanistic understanding of the action of these molecules could highlight their pleiotropic effects, strengthening their clinical results.

## 8. The Future Perspectives

Microglia and astrocytes are crucial in maintaining brain homeostasis and supporting neuronal function [15]. Microglia display a specific signature for achieving neuroprotective actions by a characteristic transcriptional profile, expression, and secretion of molecules related to their phenotypic diversity. Supporting its physiological role establishes a direct and constant cross-talk with astrocytes [7,8,9,10]. During the progression of some neurodegenerative diseases, microglia lose their homeostatic phenotype and become chronically activated. This process is characterized by gliosis and secretion of pro-inflammatory mediators (cytokines, chemokines, cytotoxic substances) that promote astrogliosis, exacerbating inflammation and damaging the CNS. Reactive astrocytes diminish their metabolic functions and collaborate in damaging BBB [11].

All GLP-1R are very safe, with few differences between them. They are well tolerated, have little and very mild secondary effects, and are suitable to be administered in the long-term as they should be therapeutically used in neuropathology [58]. These molecules emerge as a critical tool in treating neurodegenerative disease and restoring brain cell homeostasis under inflammatory conditions. Understanding the cellular and molecular targets, and whether the GLP-1R activation mechanism differs depending on sex, metabolic conditions, and stress will open new opportunities for a more personalized and safe therapy.
